# Tailoring the resonant modes in liquid crystal based all-dielectric metasurfaces

**DOI:** 10.1038/s41598-023-33693-z

**Published:** 2023-04-25

**Authors:** Pratiksha A. Sakhare, Madhunika Atmakuri, Jayasri Dontabhaktuni

**Affiliations:** 1 Department of Physics, Mahindra University, Hyderabad, India; 2 Department of Electrical Engineering, Mahindra University, Hyderabad, India

**Keywords:** Optics and photonics, Liquid crystals, Metamaterials

## Abstract

High refractive index dielectic metasurfaces are being increasingly studied for their novel light-matter interactions such as Huygen’s lens, absolute transmission and complete absorption. Liquid crystal is a versatile medium with high dielectric anisotropy and hence interaction of light with the dielectric metasurfaces immersed in liquid crystal medium show complex behaviour compared to isotropic media. Most of the investigations on liquid crystal based electromagnetic response of dielectric metasurfaces focus on tunability of resonant frequencies and switching between the resonant states as a function of external stimuli such as electric field, temperature, etc. In the current work we present a detailed numerical investigation based on studies of scattering response, near-field and far-field radiation profiles of cubic Tellurium metasurfaces as a function of liquid crystal orientations in infrared frequencies. We show that the near-field and far-field radiation profiles of primary resonant modes—electric dipoles and magnetic dipoles reorient as a function of liquid crystal orientations. In particular, we study the effect of liquid crystal orientations on novel non-radiative states called anapoles. It is observed that liquid crystal orientations effect the excitation and orientation of anapole states within the Tellurium structures. This paves way for design of an electrically-driven switch between non-radiative and radiative states. Further, controlling the near-field and far-field radiation profiles opens up possibilities in designing liquid crystal based tunable multi-functional metasurfaces which can change the directionality of incident light.

## Introduction

In sub-wavelength regime periodic dielectric structures with appropriate design can give rise to Mie responses and exhibit interesting electromagnetic properties such as negative permeability^1^, negative refractive index^2^, zero refractive index^3^, perfect absorption^4,5^ and optical chirality^6^. In plasmonic metasurfaces surface currents give rise to electric resonances and need specific geometries to exhibit magnetic resonances, where as, in dielectric structures circulating displacement currents are formed within the structures and naturally give rise to both electric as well as magnetic resonances of comparable intensities^7–9^. Such electromagnetic responses can be easily achieved even with simple geometries such as cubes, spheres or cylinders. In addition, high refractive index dielectric structures give rise to extreme localization of fields and further exhibit novel resonance modes such as non-radiative anapoles and quasi-bound states in continuum (BIC)^10–12^. Perfect BIC’s can not occur in isolated photonic structures owing to the non-existence theorem hence the significance of anapoles as the single source for non-radiative states.

Tailoring the electromagnetic response of dielectric metasurfaces have significant contributions in nanophotonics such as imaging, holography, quantum information and sensing^13,14^. A variety of tuning mechanisms are being employed to tailor the responses of dielectric metasurfaces such as phase transitions in phase change materials (PCM’s), opto-electronic and thermal tuning in liquid crystals, mechanical tuning using MEMS and microfluidic devices, etc^14–21^. Liquid crystals is a versatile medium that can support tunable, multi-functional and programmable electromagnetic response via thermal, optical, electrical and magnetic tuning^14,17,22–28^. Liquid crystal (LC) is a versatile medium with high birefringence and high dielectric anisotropy. LC’s are being increasingly investigated to design tunable, multi-functional and programmable metasurfaces^29–32^. LC medium offers controllability of opto-electronic properties as a function of external stimuli such as electric field, magnetic field, concentration and temperature^33^. LC molecules reorient their optic axis as a function of applied electric field which in turn reorients the polarization direction of incident light. Hence polarization direction of incident light and its effect on electromagnetic response of dielectric structures embedded in LC can be tuned as a function of the external electric field.

Dielectric metasurfaces embedded in liquid crystals are emerging as a major technology area with applications such as dynamic beam switching^34^, spatial light modulators^35^, metalens^36^, chiral metasurfaces^37^, electrically tunable switches^33^, color filters^38^, tunable second harmonic generation^26^, varifocal lens^39^, ultrafast switching^40^, etc. Most of the literature in liquid crystal based metasurfaces, however, focuses on the tunability of resonance frequencies as a function of LC orientations and switching between the resonant modes as a function of external fields. A comprehensive investigation of the near-field and far-field radiation patterns at these resonance frequencies as a function of LC orientations and the LC tunability of novel modes such as anapoles is absent in the literature. In the current work we perform detailed investigation of such effect of liquid crystal medium on the electromagnetic response, near-field and far-field radiation of the resonant modes.

Anapoles were first observed in the magnetic fields and has attracted considerable attention in the field of metamaterials as a possible realization of radiationless objects. In dielectric metasurfaces anapole states are formed as a result of destructive interference between electric dipole and toroidal dipoles^10,41,42^ giving rise to highly localised fields within the dielectric structures. Anapoles exhibit high Q-factors^43^ and tailoring the anapoles give rise to unprecedented applications in sensing^44,45^, non-radiative waveguides^46^, wireless power transfer^47^, high harmonic generation^48^ and non-linear optical responses^49^. Further, perfectly transmitting hybrid anapoles (HA’s) are also observed recently in high refractive index dielectric structures formed due to the destructive interference between all possible multipoles with their toroidal counterparts^50^. Anapoles in the literature are usually observed with specific designs of dielectric metasurfaces such as toroidal meta-atoms, dielectric trimers, hollow disks, etc^11,48,51–53^. In our earlier simulation study, we showed that stable anapoles can also be formed in simple structures such as high refractive index Tellurium cubes^54^. Tailoring the formation of anapoles is highly useful in the areas of communication and stealth technologies and has been achieved till now by tailoring the geometries^55^, periodicity^56^ and active materials^57^. In the current work, we numerically investigate the effect of electrically driven anisotropic LC medium on tailoring the anapole states and their orientations in near and far-field, thus opening up various possibilities in applications of programmable non-radiative modes.

## Design and simulation

**Figure 1:**
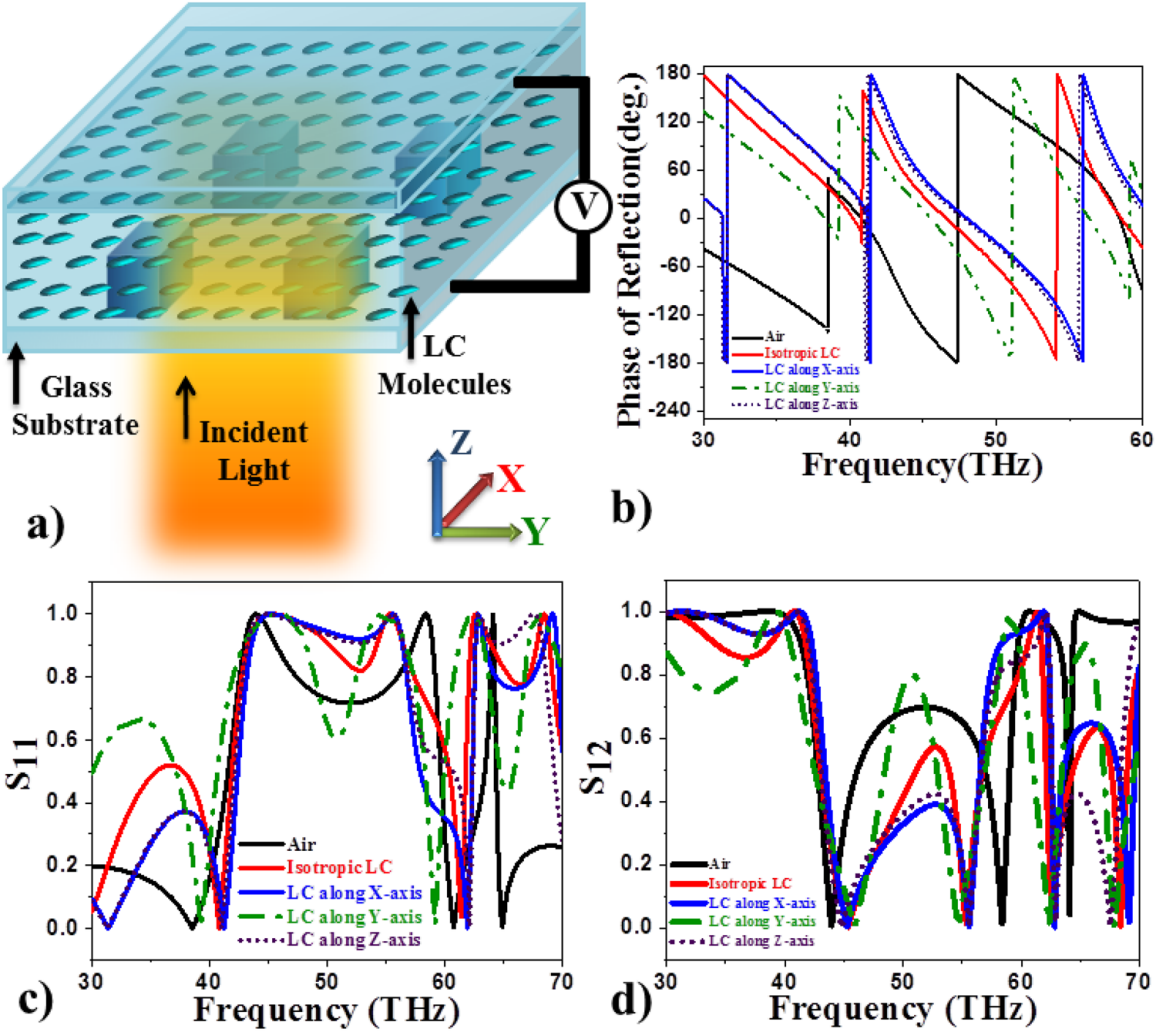
Electromagnetic response of Te metasurface in liquid crystal. (**a**) Schematic representation of cubic Te structures of dimensions 1 $\upmu$m $\times$ 1 $\upmu$m $\times$ 1 $\upmu$m with lattice constant 2 $\upmu$m introduced in nematic liquid crystal medium. (**b**) Phase of the reflected light as a function of incident frequecncies. (**c**) and (**d**) Scattering parameters $S_{11}$ and $S_{12}$ as a function of incident frequency in the range 30–70 THz, respectively.

In the present work, electromagnetic response of Te cubic structures is studied by solving Maxwell’s equations using finite element method (FEM) simulations in commercially available CST software. 2D array of Tellurium (Te) cubic resonators each with dimensions $1.0\,\,\upmu$m $\times$ $1.0\,\,\upmu$m $\times$ $1.0\,\,\upmu$m and lattice constant $2\,\,\upmu$m is placed on a glass substrate of thickness $5\,\,\upmu$m as shown in the Fig. 1a. Thickness of the cell is taken as $10\,\,\upmu$m along Z-direction and periodic boundary conditions (PBC’s) are applied along X and Y directions. Liquid crystal (LC) in nematic phase is filled between the micro-structured bottom glass plate and top glass substrate of thickness $5\mu$m. Nematic phase is characterized by zero translational order and non-zero orientational order. One can define an average direction, ’director’ along which a macroscopically large number of LC molecules orient in nematic phase. Dielectric anisotropy of liquid crystal (LC1) in nematic phase is taken as $\Delta \varepsilon =1.43$ in the current simulations^58,59^. Suppose the optic axis of LC lies in XY plane with all the molecules aligned along X-direction initially, the external electric field reorients the director axes to $\mathbf{n}=\{\cos {\alpha },\sin {\alpha },0\}$, where $\alpha$ is the angle between +X-axis and director. The permittivity tensor of the LC is then represented by

$$\begin{aligned} {\varvec{\varepsilon }}=\begin{pmatrix} \varepsilon _\perp + \Delta \varepsilon \cos ^2{\alpha } &{} \Delta \varepsilon \cos {\alpha }\sin {\alpha } &{} 0\\ \Delta \varepsilon \cos {\alpha }\sin {\alpha } &{} \varepsilon _\perp + \Delta \varepsilon \sin ^2{\alpha } &{} 0\\ 0 &{} 0 &{} \varepsilon _\perp \end{pmatrix} \end{aligned}$$

In the above matrix $\Delta \varepsilon =\varepsilon _{||} - \varepsilon _\perp$, and $\varepsilon _{||}=n_e^2$ and $\varepsilon _\perp =n_o^2$, where $n_0$ and $n_e$ are the ordinary and extraordinary refractive indices, respectively, of the LC. At higher temperatures,the dielectric permittivity in isotropic phase is expressed as $\mathbf{\varepsilon _{iso}}=\frac{2}{3}\ {\varepsilon _\perp } + \frac{1}{2}\ {\varepsilon _{||}}$.

In our simulations we considered the case in which top and bottom substrates do not induce any alignment and orientations of the LC director axes within the cell are varied in the range $0^\circ$–$90^\circ$ in XY-, YZ- and ZX-planes, respectively, as illustrated in the Fig. 1a. In practice, Te microstructures induce changes in the director field surrounding them such as topological surface defects as a result of surface anchoring and geometry, giving rise to suppression in the scalar orientational order parameter. In the current simulations we assumed very weak random surface anchoring and the topological changes in the director configuration due to geometry are ignored. Our earlier investigation on effect of geometry-induced variation in the average layer-wise director angle is of the order of $10^\circ$ near the metasurface, see^40^. Plane wave of light in the frequency range $30-70$ THz is incident on the bottom substrate along Z-axis (normal to the plane of metasurface) with polarization direction along Y-axis and H-field along X-axis. Incident light undergoes reorientation of its polarization direction along the long axis of the liquid crystal molecules as it passes through the medium. The refractive index of Te cubes is taken as 5.7($\varepsilon =33.5$ and $\mu =1$ respectively) in the frequencies considered^60^. Earlier studies on these Te cubic structures in air medium showed a reflection band in metamaterial regime with novel resonant modes such as non-radiative anapoles at 62 THz and an absolute transmission state at 60THz apart from the primary electric and magnetic dipole modes^61^.

### Electromagnetic response as a function of liquid crystal orientations

Liquid crystal molecules can be reoriented easily in XY, YZ and ZX planes by application of external aligning fields. Figure 1c and d show the scattering parameters $S_{11}$ and $S_{12}$ as a function of incident frequency in the range 30–70 THz for periodic array of Te structures in air as well as liquid crystal medium. The corresponding phases are represented in Fig. 1b. Parameters $S_{11}$ and $S_{12}$ depicted in Fig. 1c and d represent backward (reflection) and forward (transmission) scattering parameters defined as $S_{12}=Te^{(ik_0t)}$ and $S_{11}=R$, where R and T are reflection and transmission coefficients of medium, $k_0$ is the wave number in free space and *t* is the thickness of the dielectric layer, respectively. Scattering parameters exhibit a reflection band in the range 40–60 THz with two distinct peaks at both the ends in air medium^54^. Presence of nematic medium shifts the reflection band to lower frequencies (42–55 THz) with characteristic peaks varying as a function of LC orientation as shown in Fig. 1c and d. The corresponding phases undergo a variation of $2\pi$ in the reflection band as observed in Fig. 1b. In the current work we perform detailed simulations of effect of LC orientations on the electromagnetic response, near and far-field radiation patterns.

**Figure 2:**
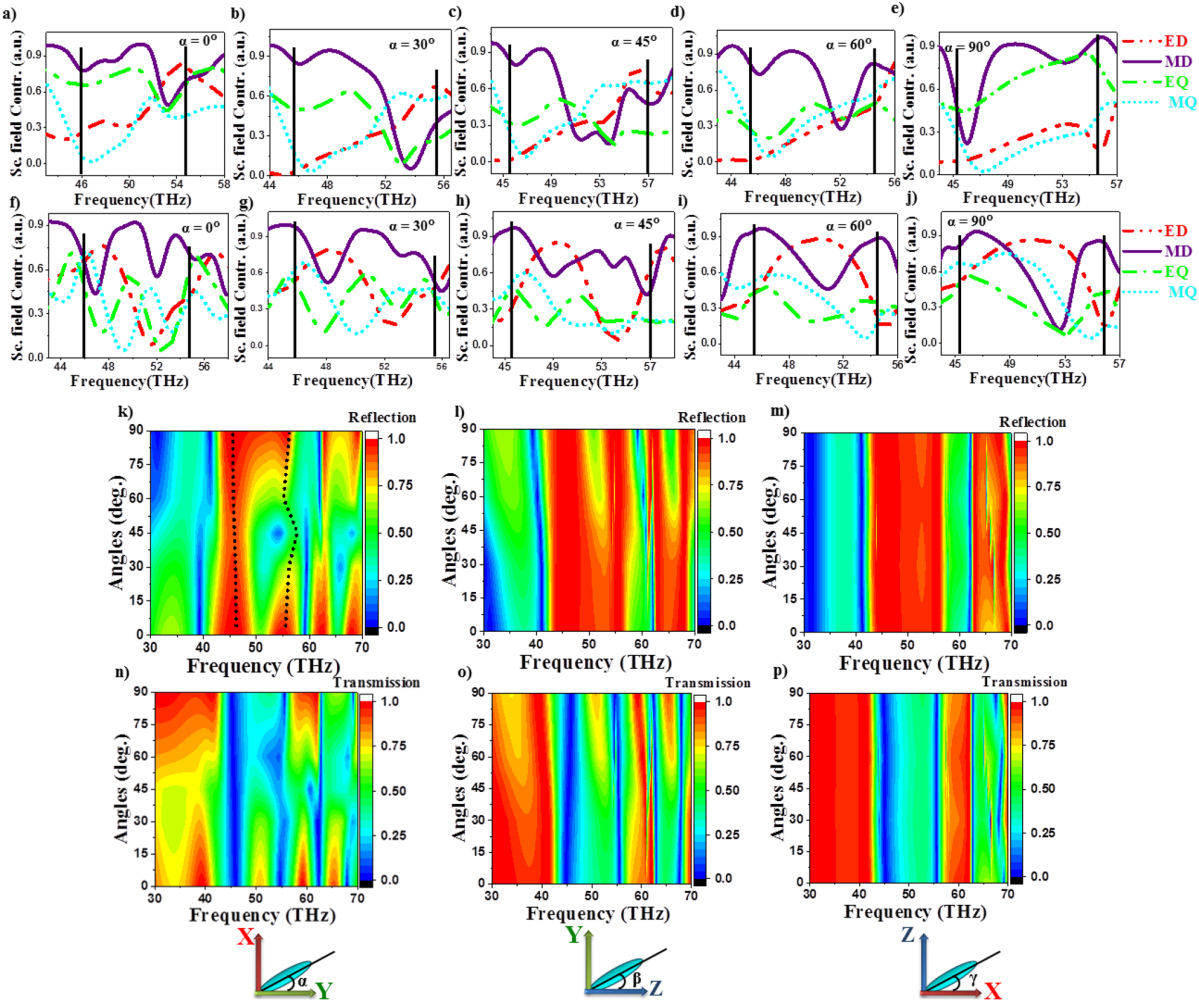
Effect of LC orientations on electromagnetic response. (**a**–**j**) Multipole contributions ED, MD, EQ and MQ for near-field distances ($1.2r_0$) (**a**–**e**) and far-field distances ($4r_0$) (**f**–**j**), respectively,  (**k**–**p**) Scattering parameters $S_{11}$ (**k**–**m**) and $S_{12}$ (**n**–**p**) as a function of incident frequencies for different LC orientations in XY, YZ and ZX planes, respectively.

At higher temperatures, LC is in isotropic phase ($\varepsilon _{iso}=3.7$ for LC1) and gives rise to an asymmetric response in the reflection band (red line in the Fig. 1c and d) in the frequency range 42–62 THz with a sharp peak on the right and broader left peak. When the LC molecules are oriented along Y-direction (incident polarization direction) leading to maximum interaction with the incident E-field, prominent and symmetric peaks are observed at both ends of the reflection band at 45 and 55 THz, respectively. LC orientations along X- and Z-axis give rise to flatter reflection bands showing an enhanced collective response from neighbouring dielectric structures.

**Figure 3:**
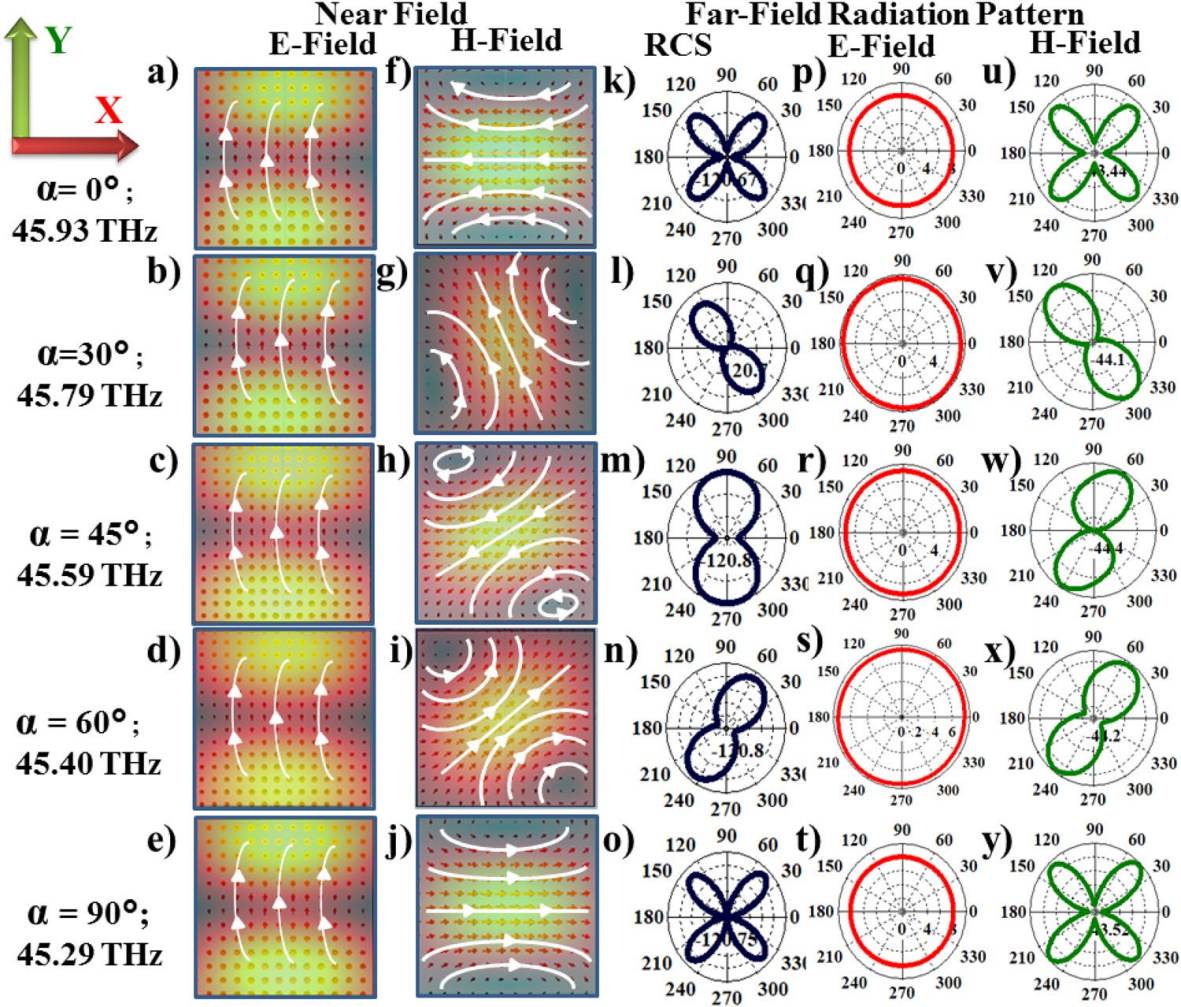
Near and far-field radiation profiles at left peaks as a function of LC reorientations in XY plane . (**a**–**j**) Near-field electric and magnetic field profiles in XY planes for LC orientations, $\alpha =0^\circ -90^\circ$ respectively. Corresponding scattered far-field radiation (**k**–**o**), electric field (**p**–**t**) and magnetic field (**u**–**y**) at the left peak frequencies, respectively.

Multipole analysis of the scattered radiation is performed to analyse the electromagnetic response obtained from the scattering parameters. In order to distinguish the contributions from toroidal ($\mathbf{T}$) and electric dipole moments ($\mathbf{p}$) we performed the analysis in Cartesian coordinate system. Multipole contributions to total scattered radiation from the total electric dipole ($\mathbf{D}$), magnetic dipole ($\mathbf{m}$), electric quadrupole (${\hat{\mathbf{Q}}}$) and magnetic quadrupole (${\hat{\mathbf{M}}}$) are obtained from the equation^62^:

$$\begin{aligned} E_{sca}=\frac{k_0^2}{4\pi \varepsilon _0}\left[ \left[ \mathbf{n}\times [\mathbf{D}\times \mathbf{n}]\right] +\frac{1}{v_d}[\mathbf{m}\times \mathbf{n}] + \frac{ik_d}{6}\left[ \mathbf{n} \times [\mathbf{n} \times {{\hat{\mathbf{Q}}}n}]\right] + \frac{ik_d}{2v_d}[\mathbf{n}\times ({\hat{\mathbf{M}}n})] \right] \end{aligned}$$

where $\mathbf{n}$ is the unit vector in the radial direction, $k_0$ is the wavenumber in vacuum, $k_d$ and $v_d$ are the wavenumber and the velocity of the light in the surrounding medium, respectively. $\mathbf{D=p}+\frac{ik_d}{v_d}{} \mathbf{T}$ is total electric dipole moment obtained from electric dipole moment $\mathbf{p}$ and the toroidal dipole moment $\mathbf{T}$. The electric dipole moment written in terms of the displacement current density $\mathbf{J}$ is $\mathbf{p}=\frac{i}{\omega }\int {\mathbf{J} dr}$, where $\mathbf{J}=-i\omega \varepsilon _0 \left[ n^2-1 \right] \mathbf{E}$ and $\mathbf{T}=\frac{1}{10c}\int {\left[ (\mathbf{r\cdot J)}r-2r^2 \right] dr}$. The definitions of higher order moments ${\hat{\mathbf{Q}}}$ and ${\hat{\mathbf{M}}}$ are followed from the reference^62^.

Multipole analysis is performed at two distances $1.2r_0$ and $4.0r_0$ representing near and far-field regimes approximately, where $r_0$ is the radius of sphere circumscribing the Te cube as shown in Fig. 2. Multipole contributions from electric dipole (ED), magnetic dipole (MD), electric quadrupole (EQ) and magnetic quadrupole (MQ) are plotted for angles $\alpha =0^\circ -90^\circ$ at both near (Fig. 2a–e) and far-field distances (Fig. 2f–j), respectively. It is observed that in case of LC orientations along Y-direction ($\alpha =0^\circ$) the left peak corresponds to MD and the right peak corresponds to ED in the near-field (Fig. 2a) while in the far-field distances, left peak corresponds to hybrid mode MD + MQ + ED and right peak corresponds to MD + EQ as shown in the Fig. 2a and f. In case of LC along X-direction ($\alpha =90^\circ$), left peak corresponds to EQ + MD and right peak corresponds to MD in near-field. At far-field distances, MD dominates at both the frequencies. For LC along Z-direction, at both near-field and far-field distances, the dominant resonant modes are same as along X-direction.

**Figure 4:**
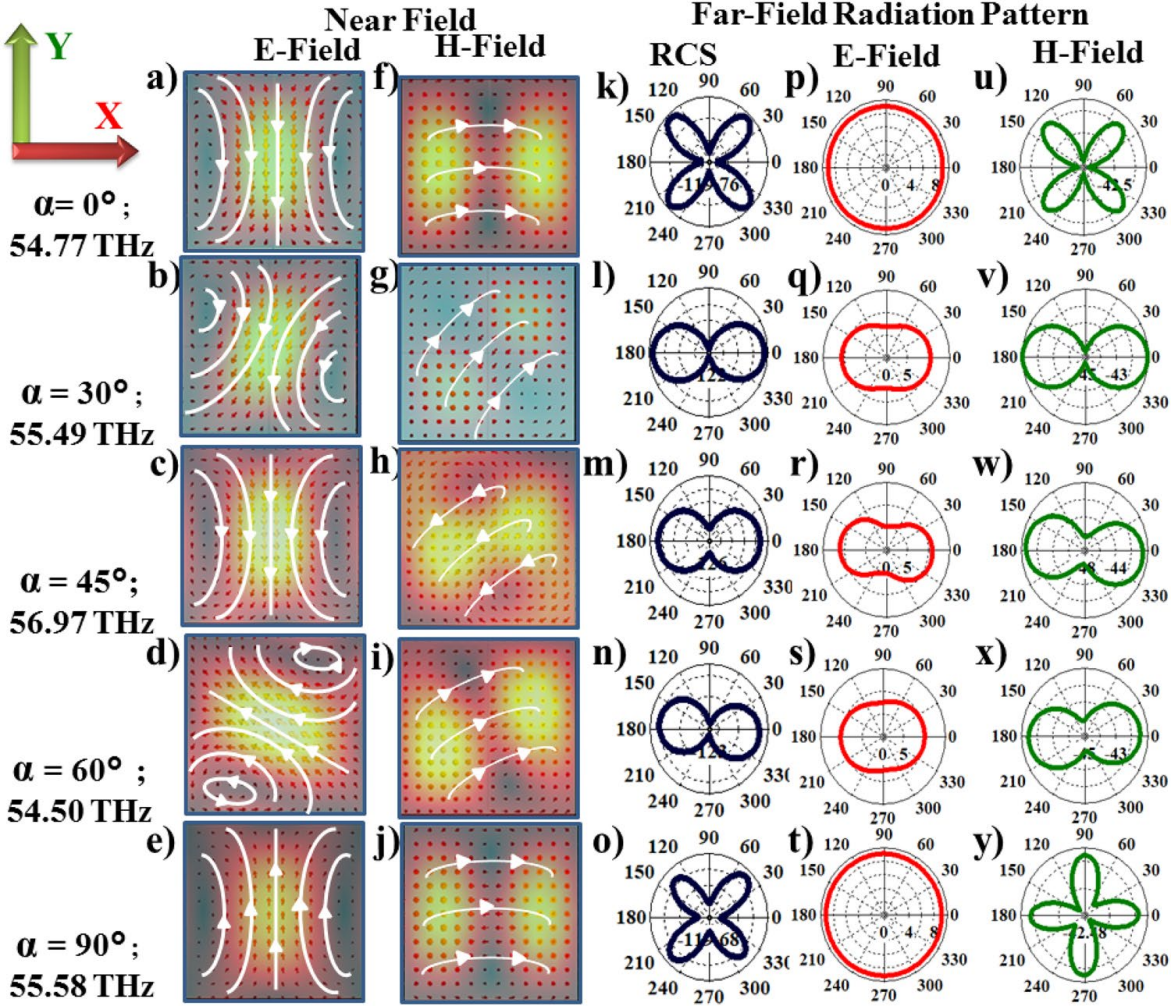
Near and far-field radiation profiles at right peak frequencies as a function of LC reorientations in XY plane. (**a**–**j**) Near-field electric and magnetic field profiles in XY planes for LC orientations, $\alpha =0^\circ -90^\circ$ respectively. Corresponding scattered far-field radiation (**k**–**o**), electric field (**p**–**t**) and magnetic field (**u**–**y**) at the right peak frequencies, respectively.

While the LC orientations along Y-axis ($\alpha =0^\circ$-incident polarization direction) exhibit both the resonant modes at 45 THz and 55 THz with equal intensity in the reflection band, it is observed that for intermediate angles ($\alpha =20^\circ -70^\circ$) the right peak is suppressed while the left peak corresponding to MD remains constant for all angles as shown in the Fig. 2k and n. This phenomenon can also be observed from the multipole analysis at far-field as shown in Fig. 2f–j. The resonance at right peak is completely suppressed at $\alpha =45^\circ$ owing to destructive interference of MD+ED modes as shown in the Fig. 2g. Hence we report that in-plane orientations of LC molecules help in selectively suppressing or enhancing a particular resonance mode giving rise to tunable opto-electronic response.

LC orientations in YZ-plane exhibit reflection band for angles $\alpha =0^\circ -45^\circ$ indicating collective response from neighboring dielectric structures. For angles $\alpha =50^\circ -90^\circ$ (closer to Y-axis) the scattering parameters show distinct left and right peaks indicating individual responses dominating over the neighboring interactions as shown in the Fig. 2l and o. In case of LC orientations in XZ plane Fig. 2m and p, scattering responses are found to be independent of the orientations of LC.

Figure 3 shows near-field (within the structures) and far- field profiles of scattered radiation at left resonance peak indicated as dashed line in Fig. 2k as a function of LC orientations. XY components of electric and magnetic field profiles within the structures, Fig. 3a–j show MD modes predominantly. We observe that these MD modes rotate within the Te cubes as a function of LC orientations in XY plane. The reorientation angles of MD modes are found to be $0^\circ$, $70^\circ , 35^\circ$, $25^\circ$ and $0^\circ$ with respect to X-axis in response to the LC orientations at $\alpha =0^\circ$, $30^\circ$, $45^\circ$, $60^\circ$ and $90^\circ$, respectively. The corresponding far-field radiation profiles—total scattered radiation (RCS), scattered electric field (E-field) and magnetic field (H-field) are shown in Fig. 3k–y. When LC orientations are along the Y-axis ($\alpha =0^\circ$) and X-axis ($\alpha =90^\circ$), far-field radiation is dominated by MQ mode. For intermediate angles MD mode dominates the far-field radiation. This can also be confirmed from multipole analysis at $r=4.0r_0$ as shown in the Fig. 2f–j. We further observed that these far-field MD modes rotate at same angles outside the dielectric structures as within them in XY plane.

**Figure 5:**
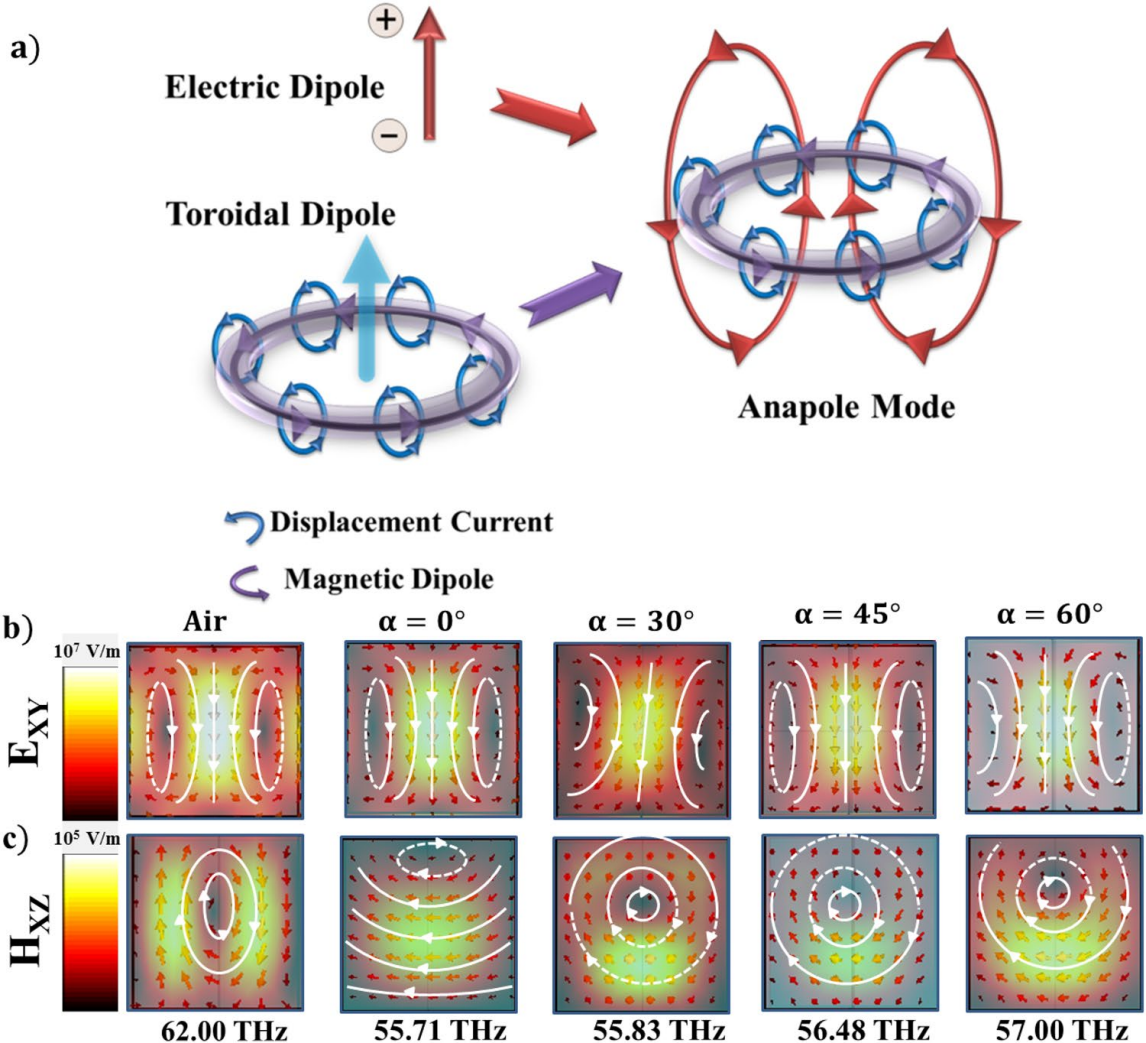
Effect of LC orientations on the excitation of anapole states. (**a**) Schematic of anapole states. (**b**) and (**c**) Near-field electric and magnetic field distributions at anapole frequencies.

**Figure 6:**
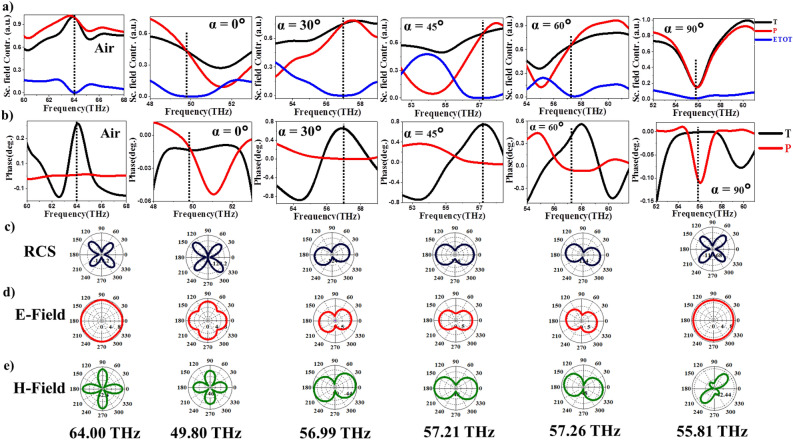
Effect of LC orientations $\alpha$ in XY plane on the far-field radiation of anapole states. (**a**) and (**b**) Multipole analysis of toroidal dipole (T), electric dipole (p) and total electric dipole (D) modes in air and LC medium with director orientations $\alpha =0^\circ$,$30^\circ$, $45^\circ$ and $60^\circ$, respectively in XY plane. (**c**–**e**) Corresponding total scattered radiation, electric and magnetic far-field radiations at anapole frequencies.

In case of right resonance peaks (indicated by dashed line in Fig. 2k) in-plane reorientations of LC ($\alpha =0^\circ -90^\circ$) give rise to rotation of ED modes within the Te structures as observed from the near-field profiles in Fig. 4. The ED’s rotate by angles $0^\circ$, $30^\circ , 0^\circ$, $45^\circ$ and $0^\circ$, respectively, with respect to Y-axis for LC orientations $\alpha =0^\circ -90^\circ$. In the case of intermediate angles $\alpha =30^\circ$, $45^\circ$ and $60^\circ$ the excitation of both MD and ED’s can be observed in the near-field profiles. Our multipole analysis at these angles showed excitation of MD and ED with comparable contributions (Fig. 2b–d). The total scattered radiation, E-field and H-field profiles in the far-field regime, Fig. 4k–y show excitation of MQ modes for $\alpha =0^\circ$ and $90^\circ$ respectively, and presence of both MD and ED modes for intermediate angles. The suppression of scattering parameters $S_{11}$ in Fig. 2k at $\alpha =45^\circ$ due to the destructive interference of ED and MD modes confirms our observations.

In the current work, we demonstrate for the first time that in dielectric metasurfaces not only the amplitude of scattering response but also the orientation of incident light can be tailored both in the near and far-field regimes at certain frequencies. Similar rotation of dominant modes can also be demonstrated for out-of-plane LC orientations. Particularly we also note here that symmetries present in the geometry of scatterers play a significant role on orientations of primary resonant modes as observed from the near- and far-field radiation.

In our earlier work on periodic Te cubic arrays we observed that radiation-less anapole states are exhibited at 62 THz in air medium^54^. In the following sections we discuss in detail the effect of in-plane liquid crystal orientations on the anapole states in nematic medium.

**Figure 7:**
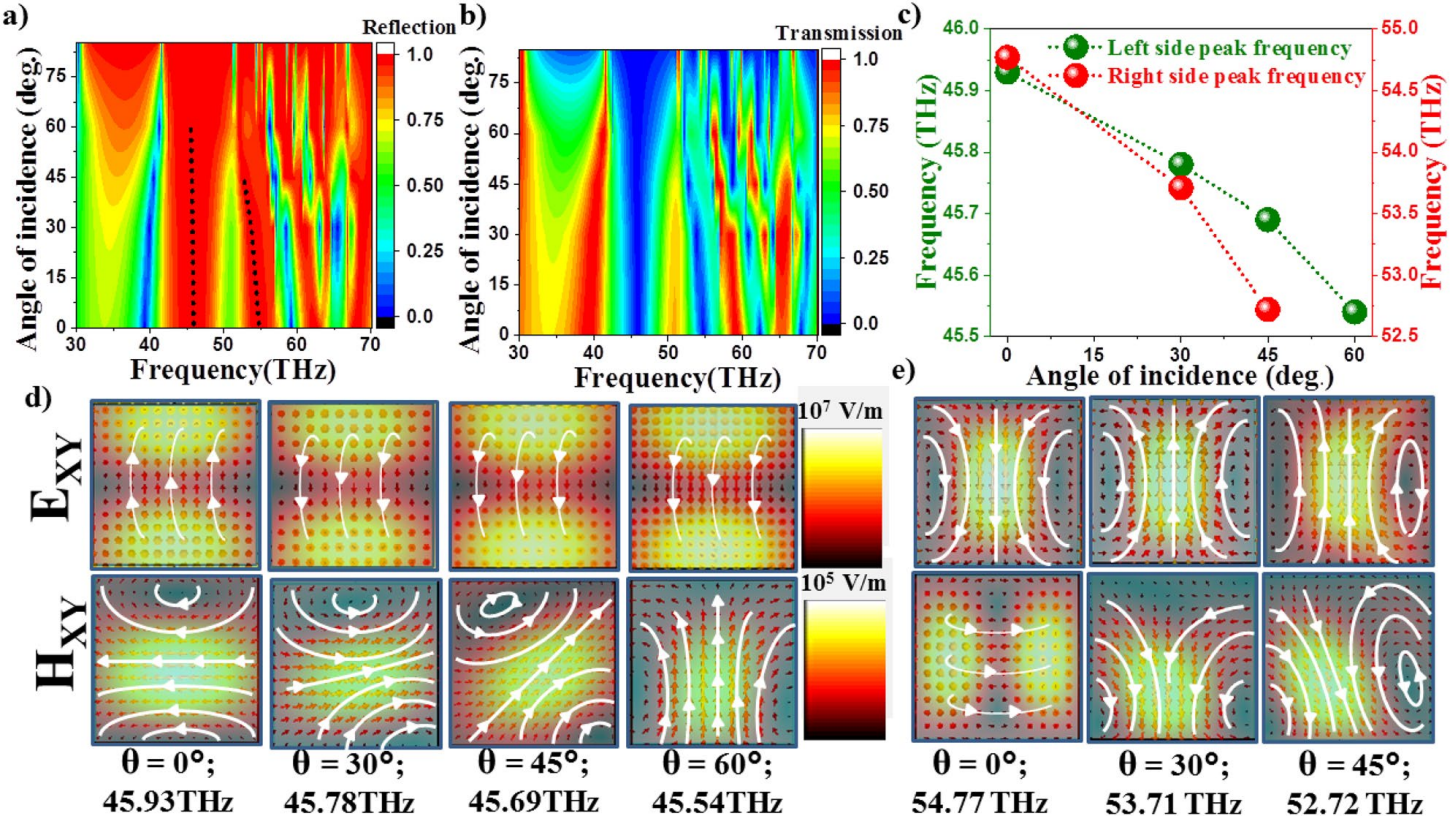
Effect of angle of incidence on the electromagnetic response of Te metasurface (**a**) and (**b**) Scattering parameters $S_{11}$ and $S_{12}$ as a function of frequency and angles of incidence, $\theta$ in the frequency range 30–70 THz respectively. (**c**) Variation of MD (left peak frequency) and ED (right peak frequency) as a function of angle of incidence. (**d**) and (**e**) Near-field electric and magnetic field profiles at MD and ED frequencies for $\theta =0^\circ -60^\circ$, respectively.

**Figure 8:**
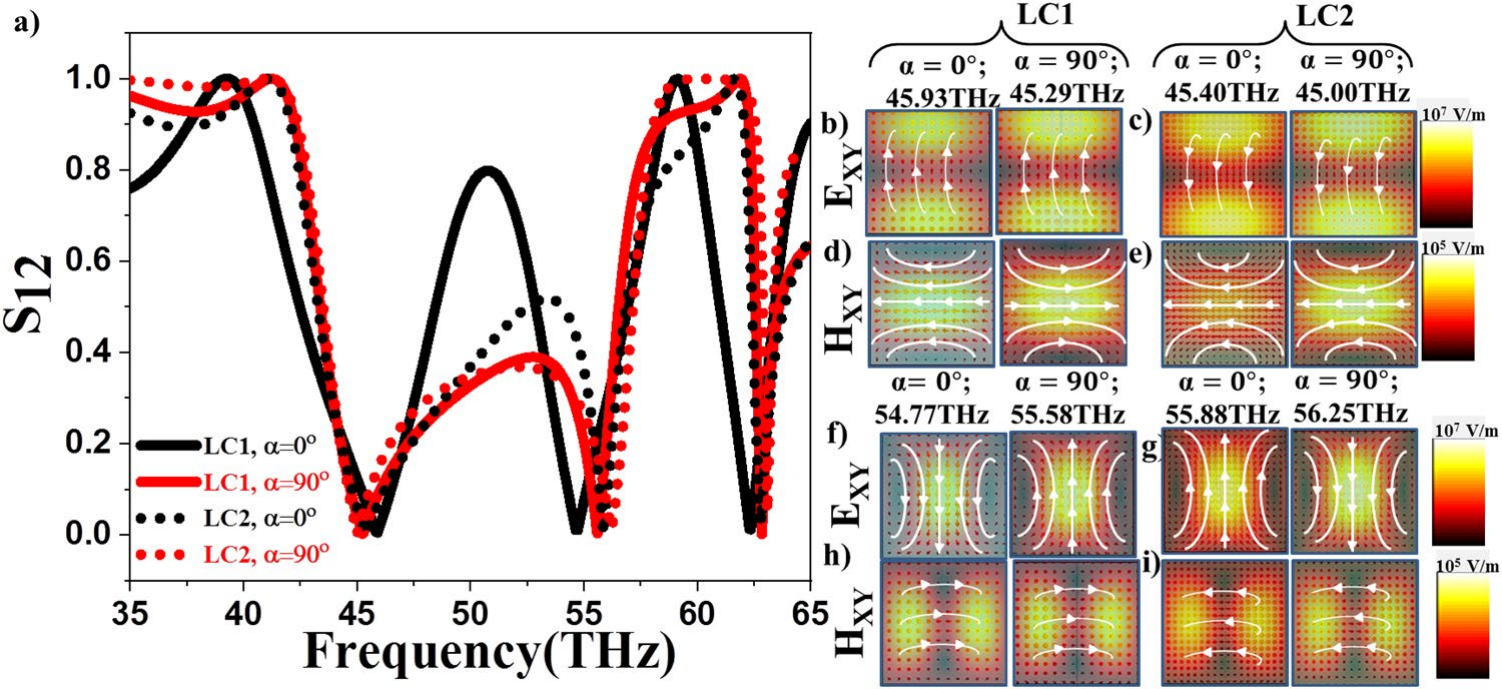
Electromagnetic response of Te structures in LC1 and LC2. (**a**) Scattering parameters, $S_{12}$ of LC1 and LC2 at LC orientations $\alpha =0^\circ$ and $90^\circ$ respectively. (**b**–**i**) Near-field electric and magnetic fields at ED and MD frequencies.

## Effect of liquid crystal orientations on anapoles

Anapoles are the non-radiative states obtained due to destructive interference between electric dipole and magnetic toroidal dipole, also called anti-Kerker effect as shown in the Fig. 5a. In the current work, we use liquid crystal medium as the active medium to induce electrically switchable anapole states in Te metasurfaces. When the Te cubes are immersed in liquid crystal medium (LC1), we observe that there is a significant effect of orientation of LC directors on the anapole directions. Near-field profiles at anapole frequencies show that anapoles orient as a function of angle of polarization (orientation of LC director) in the XY plane as shown in Fig. 5b and c. The rotation angles about Z-axis are smaller compared to the orientation angles of LC, $\alpha$. We confirm the excitation of anapoles using Cartesian decomposition of multipoles^10,63^. Scattered field contributions due to electric dipole ($\mathbf{p}$), toroidal dipole ($\mathbf{T}$) and total electric field $\mathbf{D}=ik\mathbf{T}+\mathbf{p}$ (represented as *ETOT* in the Fig. 6) at a distance $1.2 r_0$ are plotted against the incident frequencies for LC orientations $\alpha =0^\circ$, $30^\circ$, $45^\circ$ and $60^\circ$ in the Fig. 6a. The anti-Kerker condition $|\mathbf{p}| = -|ik\mathbf{T}|$ is satisfied for all LC orientations indicated by the suppression in total electric field (*ETOT*) as observed from the Fig. 6a. We further confirmed the anti-Kerker condition by plotting the corresponding phases of $\mathbf{T}$ and $\mathbf{p}$ in Fig. 6b. For all the orientations, $\alpha$, $\mathbf{T}$ and $\mathbf{p}$ have opposing phase directions. We observe that these anapole states identified by the suppression in *ETOT* (dashed lines in Fig. 6a) shift to higher frequencies as the angle of LC orientation increases. Far-field radiation profiles of anapoles in air and LC medium at angles $\alpha =0^\circ$, $30^\circ$,$45^\circ$, $60^\circ$ and $90^\circ$ are plotted in Fig. 6c–e. Total scattered radiation in air and LC medium for $\alpha =0^\circ$ and $90^\circ$ is dominated by the MQ mode as the total field due to ED’s are suppressed. For intermediate angles $\alpha =30^\circ$, $60^\circ$ and $90^\circ$, however, there is contribution from both electric and magnetic dipoles giving rise to dipolar radiation in the total scattered field oriented at different angles in XY plane. Hence presence of LC medium gives rise to non-ideal anapoles as observed both within the structures as well as the far-field. We further observe directional switching of anapoles, which was not investigated in the literature.

## Effect of angle of incidence on the electromagnetic response

We further investigated the effect of angle of incidence of incident light on the electromagnetic response of periodic Te microstructures. In these simulations polarization direction of incident light is taken along Y-axis and liquid crystal is oriented along X-axis (planar anchoring). The reflection and transmission scattering parameters are shown in the Fig. 7a and b. The left peaks of the reflection band correspond to MD and right peaks to ED for all angles of incidence. It is observed that as the angle of incidence, $\theta$ is varied from $0^\circ$ to $85^\circ$ the peaks representing dipoles (indicated by dashed lines in Fig. 7a narrow down till $\theta =60^\circ$, after which, the peaks are suppressed giving rise to broad reflection band spanning the frequency range $42-57$ THz. The MD and ED frequencies as a function of $\theta$ are shown in the Fig. 7c. It is observed that MD and ED change their orientation within the structures as observed from the near-field profiles in Fig. 7d and e respectively.

We further performed the analysis using another liquid crystal LC2 with lesser dielectric anisotropy ($\Delta \varepsilon =0.64$)^64^ given in infrared frequency range. Figure 8 shows the transmission of LC2 (dashed lines) at LC orientations $\alpha =0$ and $90^\circ$, respectively, as compared to LC1 (solid lines). It is observed that the response band is wider in case of LC2 and less pronounced in mid-transmission band indicating enhanced collective response and smaller Q-factors. Another interesting feature of LC2 is that the responses due to ED and MD are slightly an asymmetric with a sharper ED response compared to MD when the LC molecules are oriented along the polarization direction. For the LC orientations along X-direction, the response band is symmetric. This is complimentary to the response from LC1. The near field profiles in Fig. 8b–f show ED and MD resonant modes for LC2 and LC1 with LC orientations along X- and Y- directions respectively. It is observed that as the LC orientations coincide with the incident E- and H- directions the corresponding resonant modes are enhanced in both the LC’s. While the collective response of Te metasurfaces indicated by the response band is influenced by the liquid crystal chosen, the characteristics of resonant modes remain same for both the LC’s. This study helps in selecting the LC relevant for specific applications such as sensing or broad band applications.

## Discussion

In the current work, we investigated the effect of LC orientations on primary electric and magnetic responses as well as on the non-radiative anapole states. We observed that LC medium in nematic phase induces directionality to the resonant modes both within the Te structures as well as in the far-field distances as a function of orientation of LC director. A similar work undertaken^34^ shows the dynamic beam switching between isotropic and nematic phases using external electric field. In the current investigation we show that not only the far-field direction of these modes but the orientation of the modes within the structures can be switched as a function of LC orientations. Further, we showed that directionality of anapoles also change as a function of LC orientations even though they are no more completely absorptive for intermediate angles. We observed that electromagnetic response and excitation of resonant modes are strongly effected by angle of LC orientations giving rise to hybrid modes for some angles. Angle of incidence however does not affect the nature of primary resonant modes ED and MD. In the current work, we have not considered topological defects induced by dielectric structures in surrounding nematic medium and hence the heterogeneous director fields. Hence this can be approximated as LC system in the presence of strong aligning fields. In optical frequencies, the defects in nematic medium have significant effect on the electromagnetic response, which is not the case in infrared frequencies considered in the current work,

## Data Availability

The datasets used and/or analysed during the current study are available from the corresponding author on reasonable request.
